# Broad‐leaved forest types affect soil fungal community structure and soil organic carbon contents

**DOI:** 10.1002/mbo3.874

**Published:** 2019-06-19

**Authors:** Yuyu Sheng, Jing Cong, Hui Lu, Linsen Yang, Qiang Liu, Diqiang Li, Yuguang Zhang

**Affiliations:** ^1^ Key Laboratory of Biological Conservation of National Forestry and Grassland Administration Research Institute of Forest Ecology, Environment and Protection, Chinese Academy of Forestry Beijing China; ^2^ Department of Oncology The Affiliated Hospital of Qingdao University Qingdao Shandong Province China; ^3^ Shennongjia National Park Shennongjia Hubei Province China

**Keywords:** biogeochemical cycling, broad‐leaved forest, functional gene, soil fungal diversity, soil organic carbon

## Abstract

Evergreen broad‐leaved (EBF) and deciduous broad‐leaved (DBF) forests are two important vegetation types in terrestrial ecosystems that play key roles in sustainable biodiversity and global carbon (C) cycling. However, little is known about their associated soil fungal community and the potential metabolic activities involved in biogeochemical processes. In this study, soil samples were collected from EBF and DBF in Shennongjia Mountain, China, and soil fungal community structure and functional gene diversity analyzed based on combined Illumina MiSeq sequencing with GeoChip technologies. The results showed that soil fungal species richness (*p* = 0.079) and fungal functional gene diversity (*p* < 0.01) were higher in DBF than EBF. *Zygomycota* was the most dominant phylum in both broad‐leaved forests, and the most dominant genera found in each forest varied (*Umbelopsis* dominated in DBF, whereas *Mortierella* dominated in EBF). A total of 4, 439 soil fungi associated functional gene probes involved in C and nitrogen (N) cycling were detected. Interestingly, the relative abundance of functional genes related to labile C degradation (e.g., starch, pectin, hemicellulose, and cellulose) was significantly higher (*p* < 0.05) in DBF than EBF, and the functional gene relative abundance involved in C cycling was significantly negatively correlated with soil labile organic C (*r* = −0.720, *p* = 0.002). In conclusion, the soil fungal community structure and potential metabolic activity showed marked divergence in different broad‐leaved forest types, and the higher relative abundance of functional genes involved in C cycling in DBF may be caused by release of loss of organic C in the soil.

## Graphical abstract

Evergreen broad‐leaved (EBF) and deciduous broadleaved (DBF) forests soil samples were collected and soil fungal community structure and functional gene diversity were analyzed combined Illumina MiSeq sequencing with GeoChip technologies. The results showed the soil fungal community structure and potential metabolic activity had marked divergence in different broadleaved forest types, and the higher relative abundance of genes involved in C cycling in DBF caused soil organic C release or loss of the soils.

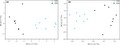

## 1 INTRODUCTION

Broad‐leaved forests are one of the most common and important forest types in terrestrial ecosystems. There are numerous evergreen broad‐leaved forests (EBF) and deciduous broad‐leaved forests (DBF) in subtropical China (Ding et al., 2015). These forest ecosystems are known for both their quantity and quality of plant leaf litter (Guo et al., 2016), which greatly impacts underground organic matter (Yang et al., 2009) and soil microbial diversity (Ding et al., 2015; Hu, Jin, Liu, & Yu, 2014). In recent years, careful attention has been paid to the impacts of broad‐leaved forest conversion on plant species diversity (Huang et al., 2015) and soil labile organic matter (Chen et al., 2016; Yang et al., 2009), as well as differences in plant photosynthetic activity (Villar, Robleto, Jong, & Poorter, 2006) and soil nutrient requirements (Aerts, 1995; Givnish, 2002) between EBF and DBF. However, underground microbial diversity and their associated metabolic activities of EBF and DBF are poorly understood, particularly the soil fungal composition and related functional gene diversity.

Soil fungi play critical and unique roles in terrestrial ecosystem processes, such as regulation of the carbon (C) cycle, decomposition of plant litter, and monitoring of soil pathology (Hawkes et al., 2011; Penton et al., 2013; Tedersoo et al., 2014; Yang, Adams, et al., 2017). However, few studies have investigated soil fungi owing to the limited technologies surrounding culture and morphological identification. With the development of high‐throughput sequencing technology over the last few years, soil fungi have received extensive attention, particularly with respect to variations along different spatial scales and environmental gradients, such as temperature (Zhou et al., 2016), precipitation (Hawkes et al., 2011; Zumsteg, Bååth, Stierli, Zeyer, & Frey, 2013), pH (Rousk et al., 2010), organic phosphorus (Bao et al., 2013), organic C (Hanson, Allison, Bradford, Wallenstein, & Treseder, 2008), and anthropogenic disturbances (Sun et al., 2015; Wang, Song, et al., 2017). Plant diversity has a strong correlation with soil fungi at local scales (Tedersoo et al., 2016; Yang, Adams, et al., 2017) but a weak correlation at global scales (Tedersoo et al., 2014). This difference in correlation strength may be due to climatic factors that overshadow plant influence at global scales (Bahram, Põlme, Kõljalg, Zarre, & Tedersoo, 2012).

Soil labile fractions play vital roles in maintaining soil fertility and providing adaptability to environmental effects (Yang et al., 2009). Natural climate change and anthropogenic activity can impact belowground quantity of soil organic C (SOC) (Poeplau & Don, 2013; Song, Kimberley, Zhou, & Wang, 2016; Zhang et al., 2017). The effects of vegetation conversion on SOC have been focused on changes related to forest age, quantity and quality of litter fall, soil disturbance (Song et al., 2016; Yang et al., 2009) and dominance of soil microbes with various strategies (Chen et al., 2016). Previous studies have shown that microbial activity is related to C cycling based on investigations of microbial efficiency (Frey, Lee, Melillo, & Six, 2013; Six, Frey, Thiet, & Batten, 2006), metabolic quotient model (Bini et al., 2013), soil respiration (Carey et al., 2016; Song et al., 2016), phospholipid fatty acid analysis, and extracellular enzyme activity (Smith, Marín‐Spiotta, & Balser, 2015; Smith, Marín‐Spiotta, Graaff, & Balser, 2014). Studies have shown a direct correlation between soil bacterial functional gene diversity and SOC content (Xue et al., 2016; Zhang et al., 2017) based on GeoChip technology (Cong, Liu, et al., 2015; Tu et al., 2014; Yang et al., 2014; Zhang, Cong, et al., 2014). However, little is known about specific bioprocess of SOC fractions related to soil fungal diversity.

To understand the soil fungal community structure and functional gene diversity in broad‐leaved forests, soil samples from EBF and DBF in Shennongjia National Reserve (SNNR) were collected and analyzed using both Illumina sequencing and a microbial functional gene array (GeoChip 4.0). SNNR is well known in China for its extraordinarily rich biodiversity (Ma et al., 2008). The specific goals of this study were as follows: (a) to determine the soil fungal taxonomic and functional gene community structure and the differences between EBF and DBF; (b) to identify the linkage between soil fungal functional genes involved in soil C and nitrogen (N) cycling and soil nutrients; and (c) to explore the key environmental factors shaping soil fungal community structure in broad‐leaved forests.

## 2 MATERIALS AND METHODS

### 2.1 Study sites and soil sampling

Sampling sites were located in the SNNR, northwest Hubei Province, China. The study area has a mean annual temperature of 7.2°C and a mean annual precipitation of 1,500 mm (Ma et al., 2008). The EBF was located at 31°24ʹN, 110°20ʹE and the DBF at 31°29ʹN, 110°21ʹE. Eight study plots (20 m × 20 m) were established in each forest type with a distance of over 20m between contiguous plots. In each plot, 10–15 soil cores were collected (0–10 cm depth) and mixed. Stones and plant roots were removed by sieving through a 2 mm mesh. The mixed soil samples were divided into two parts. One part was stored at −80°C for DNA extraction and the other at 4°C for measurement of soil physicochemical parameters.

### 2.2 Soil physicochemical parameters and plant survey

The soil temperature of each plot was measured using a long‐stem thermometer (SPECTRUM, USA) at a depth of 10 cm. The SOC, dissolved SOC (DSOC), labile SOC (LSOC), total nitrogen (TN), total phosphorus (TP), available nitrogen (AN), available phosphorus (AP), pH, and moisture (Mo) were measured (Bao, 2000). SOC and TN were measured using the wet oxidation and a modified Kjeldahl procedure, TP was determined using a wet digestion method with concentrated HF and HClO_4_, and soil pH was measured at a water to soil ratio of 2.5:1 using a pH meter with a glass electrode (Cong, Yang, et al., 2015). Soil Mo was detected by weighing after drying in an oven at 105°C for 10 hr (Cong, Yang, et al., 2015). The mean annual temperature and mean annual precipitation were obtained from IPCC5 (http://www.worldclim.org). Plant properties in each plot were surveyed, including plant species, tree number, canopy, height, and diameter at breast (1.3 m).

### 2.3 DNA extraction, purification, and quantification

Soil microbial DNA was extracted using the E.Z.N.A Soil DNA Kit (OMEGA BioTek, USA) according to standard protocols. Soil microbial DNA quality and concentration were assessed by ratios of absorbance at 260 nm/280 nm (1.8–2.0) and 260 nm/230 nm (>1.7) using a NanoDrop ND‐1000 Spectrophotometer (NanoDrop, Wilmington, DE).

### 2.4 Soil fungal Illumina sequencing and data processing

The internal transcribed spacer II (ITS2) of soil fungi ribosome encoding genes was amplified using the primers: gITS7F (5′‐GTGARTCATCGARTCTTTG‐3′) and ITS4R (5′‐TCCTCCGCTTATTGATATGC‐3′) (Ihrmark et al., 2012). The barcode sequence for each sample was designed and combined with the reverse primer. Polymerase chain reaction (PCR) amplification was performed in a 50 μl reaction consisting of 5 μl 10× Taq Buffer, 1.5 μl dNTP, 0.5 μl Taq (Tiangen, Beijing, China), 2 μl BSA (5mg/ml), 1 μl each primer, 1 μl soil microbial template DNA (~25 ng/μl), and 38 μl ddH_2_O. The mixtures were amplified under the following conditions: initial denaturation at 94°C for 1 min, followed by 35 cycles of 94°C for 20 s, 56°C for 25 s, and 68°C for 45 s, with final extension at 68°C for 10 min.

Polymerase chain reaction products were separated using electrophoresis of 1.5% agarose gels and purified using the E.Z.N.A gel extraction kit (Omega, Georgia, USA). Products were mixed according to postpurification concentration and optical density. The mixed products were quantified using Qubit dsDNA HS standard (0 ng/ml DNA) and Qubit dsDNA HS standard (500 ng/ml DNA). VAHTSTM PCR‐Free DNA Library Prep Kit for Illumina was used to construct the cDNA library (Zhou et al., 2016). Once constructed, the DNA library was denatured at 96°C for 2 min and the PhiX Control Library added. Libraries were then kept in an ice‐water mixture for 5 min. Finally, 600 μl of the reaction mixture was injected into the MiSeq Reagent cartridge (Illumina, San Diego, CA) for paired‐end 250 bp sequencing (Zhou et al., 2016).

Raw sequences were preprocessed using the Galaxy pipeline (http://mem.rce es.ac.cn:8080). Sequences of different barcode primers were trimmed, after which reads from the same sequence were combined using FLASH (Magoč & Salzberg, 2011) and Btrimmed (average quality score >20; window size = 5) (Zhou et al., 2016). Sequences with an ambiguous base (including “N”) or those less than 200 bp were deleted (Zhou et al., 2016). Chimeric sequences were removed using prediction algorithms in Uchime (Edgar, Haas, Clemente, Quince, & Knight, 2011). UCLUST was performed to classify operational taxonomic units (OTUs) at a 0.97 threshold. Random resampling was achieved with 10,000 sequences per sample. Taxonomic assignment was performed using the Ribosomal Database Project classifier with 50% confidence through the fungal ITS UNITE database (Wang, Li, et al., 2017; Zhao et al., 2016).

### 2.5 GeoChip hybridization and data processing

DNA hybridization was performed using GeoChip 4.0, which contains 4,965 oligonucleotide probes from 127 Eukaryotic microbial gene categories involved in C and N cycling and other biogeochemical processes (Tu et al., 2014). Purified microbial DNA was labeled with Cy5 fluorescent dye using a random priming method, and GeoChip hybridization carried out at 45°C for 10 hr with 50% formamide (Zhang, Cong, et al., 2014). The hybridized GeoChip was scanned (Perkin‐Elmer, Wellesley, MA) and quantified based on signal intensity using ImaGene 6.0 (Biodiscovery, El Segundo, CA) (Zhang, Cong, et al., 2014).

GeoChip data were preprocessed by: (a) deleting spots for which there was a signal‐to‐noise ratio of less than 2.0 or a signal intensity less than 1,000; (b) removing genes detected in no more than three out of eight samples from the same site; (c) natural log converting; and (d) dividing by each mean value of each slide.

### 2.6 Soil fungal functional gene molecular ecological network construction

Functional molecular ecological networks (fMENs) were established using soil fungal functional genes related to C degradation to illustrate the links of nodes. Environmental factors and selected genes detected in less than 8 of 16 samples were removed to identify the linkage between networks and variables (Deng et al., 2012). To ensure that identification was reliable, sensitive and robust, thresholds of network structure analysis were selected mathematically by the random matrix theory (RMT)‐based method (Deng et al., 2012; Zhou et al., 2010). Empirical and random network properties were obtained from the Molecular Ecological Network Analysis Pipeline of the Institute for Environmental Genomics (http://ieg2.ou.edu/MENA), and data were further visualized using the Cytoscape 3.4.0 software.

Each node plays a different role in fMENs. Roles were defined by parameters of within‐module connectivity (*Zi*), which reveals connectivity of nodes from one to another in the same module, and module connectivity (*Pi*) which reveals the connectivity of nodes with other modules (Olesen, Bascompte, Dupont, & Jordano, 2007). According to the value of *Zi* and *Pi*, nodes were classified into four groups using the described classification standard: (a) peripherals (*Zi* ≤ 2.5, *Pi* ≤ 0.62), which are nodes that often connect to nodes in their own module that have less connectivity, (b) connectors (*Zi* ≤ 2.5, *Pi* > 0.62), which are nodes that highly connect with several modules, (c) module hubs (*Zi* > 2.5, *Pi* ≤ 0.62), which are nodes that highly connect with nodes in their own module, and (d) network hubs (*Zi* > 2.5, *Pi* > 0.62), which are nodes that act as both module hubs and connectors (Deng et al., 2012).

### 2.7 Statistical analysis

Plant diversity was represented by the Simpson index. Soil fungal diversity was represented by the number of detected soil fungal OTUs (soil fungal ITS2 richness) from Illumina sequencing and the soil fungal functional genes from GeoChip 4.0. An unpaired *t *test was performed to identify differences between the two parameters and abundance. Detrended correspondence analysis (DCA) and a dissimilarity test based on Bray–Curtis and the Euclidean distance were performed to identify differences in fungal community composition and structure, respectively. Pearson correlation analysis, mantel test, and canonical correlation analysis (CCA) were used to identify the major environmental factors impacting soil fungal diversity. Factors used for the CCA model were selected by retaining variance inflation factors of less than 20 to remove redundant factors that had interfered with others (He et al., 2010; Zhao et al., 2016). All statistical analyses were conducted using the R package vegan (v.3.5.2), Institute for Environmental Genomics online platform (http://ieg.ou.edu/) and IBM SPSS Statistics (V.21.0). Visualization of the data was conducted using SigmaPlot 12.5.

## 3 RESULTS

### 3.1 Soil physicochemical parameters and plant diversity

Soil physicochemical properties and plant diversity were analyzed (Table 1). Most of the SOC and soil nutrients were significantly lower (*p* < 0.05) in DBF than EBF, such as SOC (*p* = 0.049), LSOC (*p* = 0.03), TN (*p* = 0.008), AN (*p* = 0.007), and TP (*p* = 0.048). According to the plant survey, the dominant plant species were *Machilus calcicola*, *Styrax suberifolius*, and *Cyclobalanopsis gracilis* in EBF and *Quercus aliena*, *Carpinus viminea,* and *Fagus engleriana* in DBF. Plant diversity was significantly higher (*p* < 0.05) in EBF than DBF. Therefore, most soil physicochemical parameters and plant diversity significantly differed between EBF and DBF.

**Table 1:** Summary of plant diversity, soil fungal diversity, and soil chemistry parameters (*n* = 8)

Parameters	EBF	DBF	*P* value (unpaired‐*t* test)
Plant Simpson index	14.22 ± 4.07	7.63 ± 5.83	0.020
Soil fungal ITS richness	532.13 ± 172.22	749.38 ± 275.32	0.079
Soil fungal functional gene richness	3,000.13 ± 317.96	3,633.75 ± 508.77	0.010
Soil organic carbon (g/kg)	52.58 ± 28.88	28.24 ± 2.89	0.049
Dissolved soil organic carbon (g/kg)	0.25 ± 0.16	0.14 ± 0.05	0.082
Labile soil organic carbon (g/kg)	7.97 ± 4.20	1.28 ± 0.51	0.003
Total nitrogen (g/kg)	4.14 ± 1.80	1.83 ± 0.33	0.008
Available nitrogen (mg/kg)	286.19 ± 95.92	173.33 ± 28.93	0.007
Total phosphorus (g/kg)	1.20 ± 1.15	0.23 ± 0.03	0.048
Available phosphorus (mg/kg)	6.17 ± 2.85	3.26 ± 0.37	0.023
Soil pH	6.58 ± 0.91	5.35 ± 0.53	0.005
Soil moisture	0.37 ± 0.07	0.49 ± 0.05	0.002
Soil temperature at the 10 cm depth (°C)	19.82 ± 0.52	16.56 ± 0.33	<0.001
Mean Annual precipitation (mm)	1,067	1,234	
Mean Annual temperature (°C)	12.7	9.5	

Note

Data were presented in mean value and standard error, *P* value of each parameter was measured by unpaired *t *test.

### 3.2 Soil fungal diversity and community composition

A total of 6,399 soil fungal ITS2 OTUs were obtained in DBF and EBF by Illumina sequencing. The number of sequences per sample ranged from 10,743 to 130,335. After conducting 10,000 resampling per sample, 2,316 and 2,827 OTUs were obtained for EBF and DBF, respectively.

At the phylum level, soil fungal ITS2 OTUs were classified into five phyla, *Zygomycota, Ascomycota*, *Basidiomycota*, *Chytridiomycota,* and *Glomeromycota*. The dominant phylum in both forest sites was *Zygomycota* (53.79% in EBF and 58.13% in DBF), followed by *Ascomycota* (36.24% in EBF and 23.75% in DBF) and *Basidiomycota* (8.70% in EBF and 17.64% in DBF). At the genus level, a total of 267 genera were identified (Table A1). Among all genera, *Umbelopsis* had the highest relative abundance in DBF (49.20%) and *Mortierella* had the highest relative abundance in EBF (44.07%). The relative abundance of different genera differed significantly (*p* < 0.05) between EBF and DBF (Table A1).

**Figure 1:**
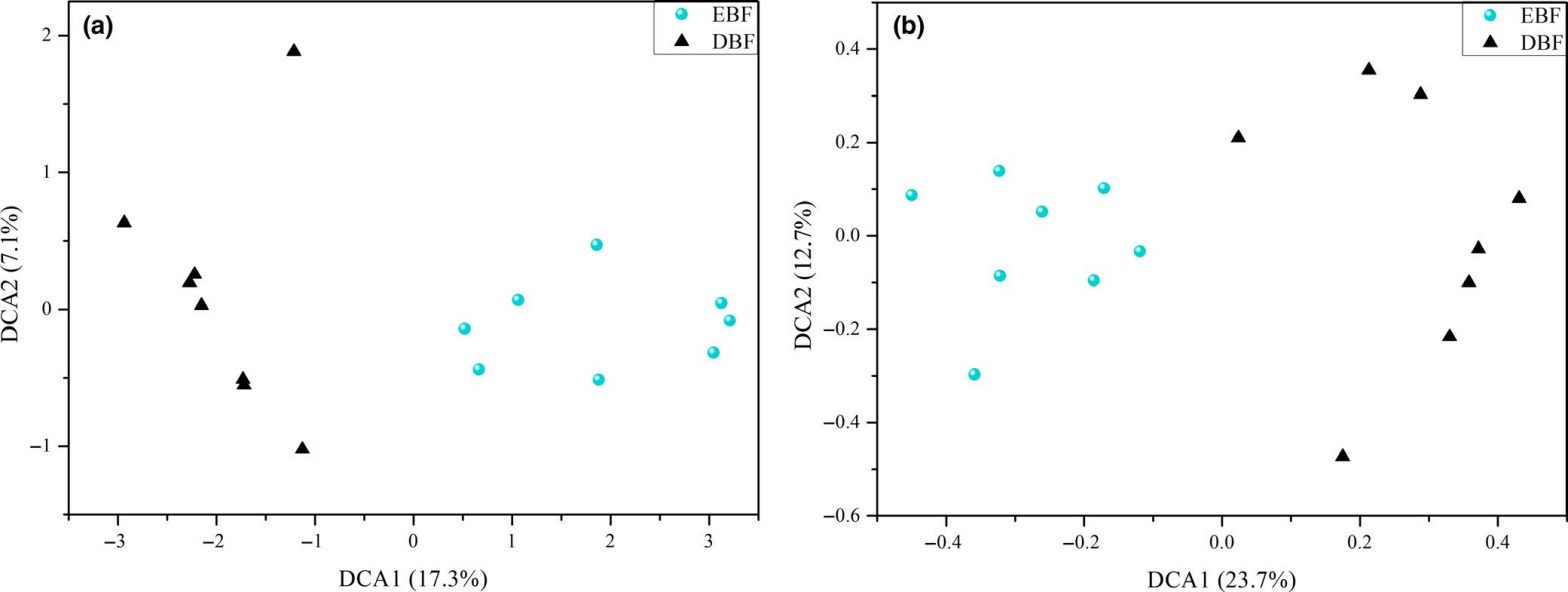
Detrended correspondence analysis (DCA) of soil fungal community. The data were analyzed based on ITS2 OTUs from the Illumina sequencing (a) and relative signal intensity of soil fungal functional genes from GeoChip 4.0 (b)

Soil fungal ITS2 richness was higher (*p* = 0.079) in DBF than EBF (Table 1). DCA indicated that soil fungal communities were well separated from each site (Figure 1a). Dissimilarity tests were conducted using different permutation tests (MRPP, ANOSIM, and Adonis) and significant differences (*p* < 0.01) were found between the two sites (Table A2).

### 3.3 Soil fungal functional genes involved in C and N cycling

A total of 4,439 soil fungal functional gene probes were detected in two broad‐leaved forest sites (3,589 in EBF and 4,371 in DBF). Fungal functional gene diversity was significantly higher (*p* < 0.01) in DBF than EBF (Table 1). DCA indicated that soil fungal gene communities were well separated from each site (Figure 1b).

**Figure 2:**
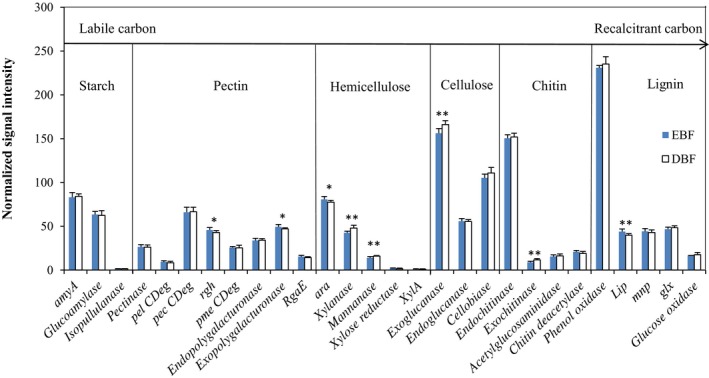
The normalized average signal intensity of key gene categories involved in C degradation. The signal intensity of each gene is an average of eight samples after transferring into logarithm and dividing by the mean value of each slide. Each bar is presented as mean and standard error (*n* = 8). Significant differences are denoted by *(*p* < 0.05) or **(*p* < 0.01) above corresponding bars

A total of 2,215 fungal gene probes involved in C cycling were detected, including C 2,075 degradation genes related to lignin, cellulose, pectin, chitin, starch and hemicellulose, and 140 C fixation genes. The relative abundance of many genes related to labile C degradation (starch, pectin, hemicellulose, and cellulose) was significantly higher (*p* < 0.05) in DBF than EBF (Figure 2). For example, the *xylanase* and *mannanase* genes involved in hemicellulose degradation, *exoglucanase* gene involved in cellulose degradation and *exochitinase* gene involved in chitin degradation were significantly higher (*p* < 0.01) in DBF than EBF. However, the relative abundance of the *chitin synthase* gene, which was the only gene involved in C fixation that was detected, was significantly lower (*p* = 0.027) in DBF than EBF. Moreover, the relative abundance of C cycling genes was significantly negatively correlated with soil SOC (*r* = −0.580, *p* = 0.018), DSOC (*r* = −0.508, *p* = 0.044) and LSOC (*r* = −0.720, *p* = 0.002).

**Figure 3:**
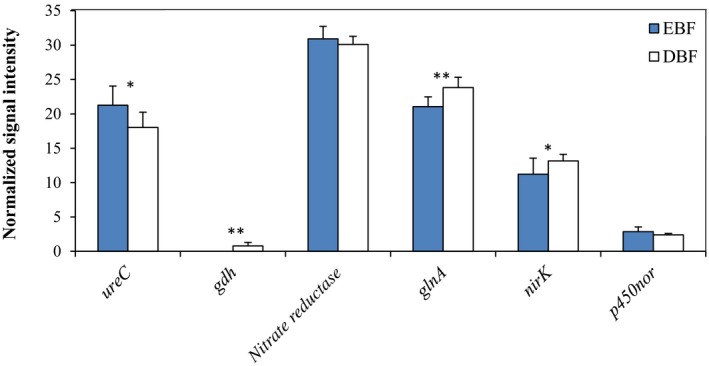
The normalized average signal intensity of key gene categories involved in N cycling. The signal intensity of each gene is an average of eight samples after transferring into logarithm and dividing by the mean value of each slide. Each bar is presented as mean and standard error (*n* = 8). Significant differences are denoted by *(*p* < 0.05) or **(*p* < 0.01) above corresponding bars

A number of functional genes involved in N cycling were detected at the two sites, including the *ureC* and *gdh* genes related to ammonification, *nitrate reductase,* and *glnA* genes related to assimilatory N reduction, and the *nirK* gene related to denitrification. The relative abundance of the *gdh*, *glnA,* and *nirK* genes was significantly higher (*p* < 0.05) in DBF than EBF (Figure 3). Pearson's correlation analysis showed that the relative abundances of *gdh*, *glnA*, *nitrate reductase,* and *nirK* genes were negatively correlated with soil TN and AN, particularly for *gdh* (Table A3). Therefore, the soil fungal functional gene community differed significantly between the two broad‐leaved forests.

### 3.4 Ecological network analysis of soil fungal functional genes

**Figure 4:**
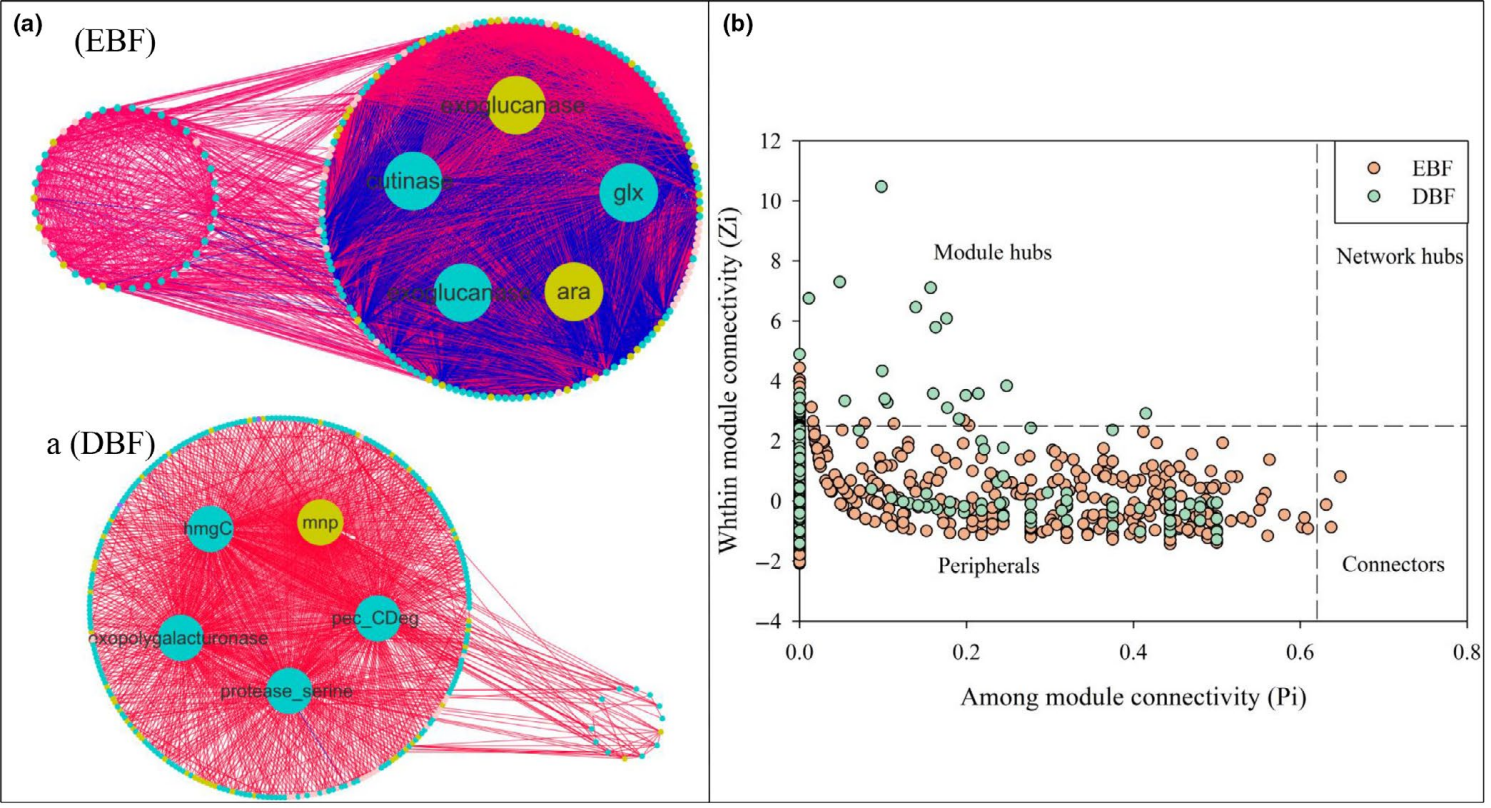
The submodules of functional molecular ecological networks related to C degradation genes. (a) The first five nodes with high connectivity and their neighbors at evergreen broad‐leaved forest (EBF) and deciduous broad‐leaved forest (DBF). Edges between each pair of nodes represent different interactions, where edges in blue indicate positive interactions and edges in red indicate negative interactions. (b) Z‐P plot presenting the four roles of genes defined in terms of their values among module connectivity (*Pi*) and within module connectivity (*Zi*), Z‐P plot was drawn by carbon degradation functional genes, dots represent selected genes of evergreen broad‐leaved forest (in orange) and deciduous broad‐leaved forest (in green)

A total of 1,428 genes in EBF and 1,317 genes in DBF were selected to establish fMENs, of which 902 were shared between the two networks (Table A4). The modularity was higher in the DBF network (0.794) than the EBF network (0.620). Calculation of the positive percentage of edges of each network revealed that 93.37% of the positive interactions existed in the DBF network and 73.93% existed in the EBF network. The top five nodes with high connectivity (Table A5) and their linked neighbors were selected to draw a subunit network (Figure 4a). There were no nodes in common, and the EBF network showed a large amount of negative interactions when compared with the DBF network.

The Z‐P plot was classified into four groups based on the value of *Zi* and *Pi* (Figure 4b). The majority of nodes were shown as peripherals. A total of 23 and 31 module hubs were detected in EBF and DBF, respectively (Table A6). No node IDs were shared between the two networks. There was no connector in DBF, while there were three connectors in EBF, including one cellulose degradation gene (*exoglucanase*), one pectin degradation gene (*rgh*) and one starch degradation gene (*amyA*), and the relative abundance of *exoglucanase* (*p* < 0.01) and *rgh* (*p* < 0.05) differed significantly between the two sites. Finally, there was no network hub in either site.

Overall, the key genes of soil fungal fMENs related to C degradation were significantly different between two broad‐leaved forest types, and the modularity and percentage of positive interactions was significantly higher in DBF than EBF.

### 3.5 Linkage between soil fungal community and environmental factors

**Figure 5:**
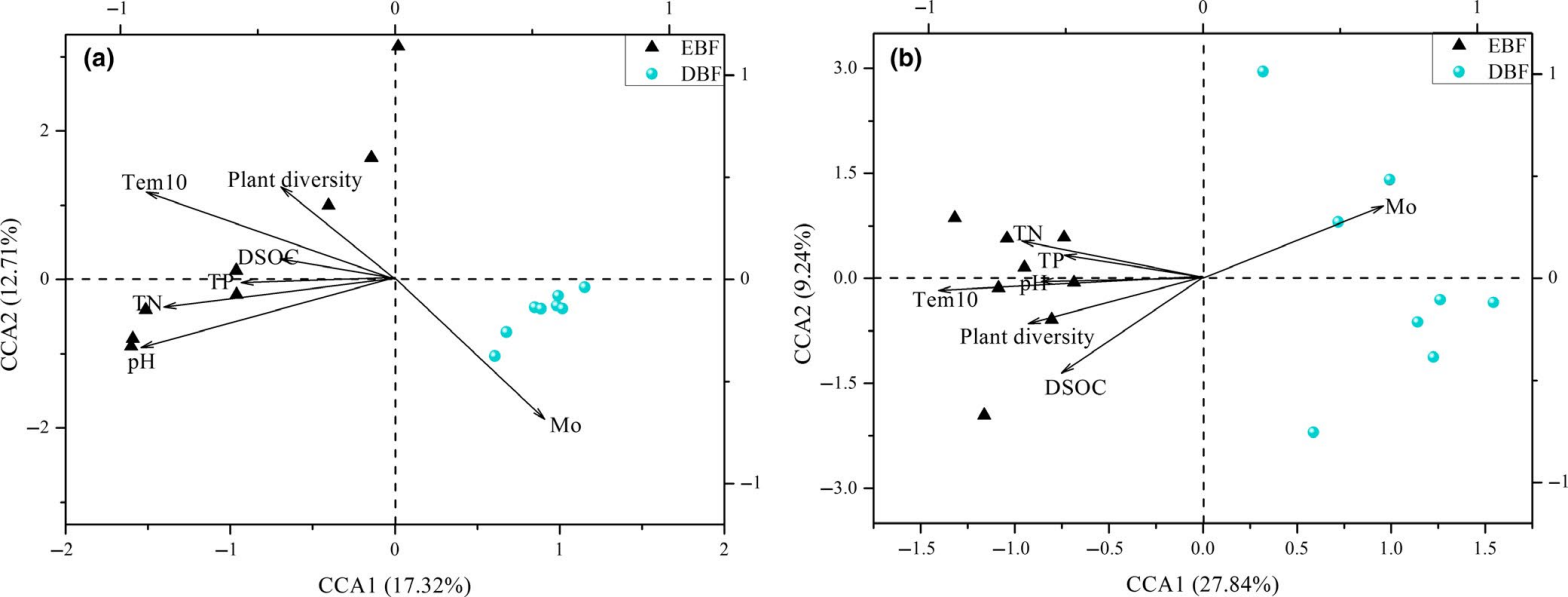
Canonical correspondence analysis (CCA) of soil fungal community and functional genes at two broad‐leaved forest types. The CCA was analyzed based on soil fungal ITS2 OTUs (a) and relative signal intensity of soil fungal functional genes based on GeoChip 4.0 (b). Note: EBF, evergreen broad‐leaved forest; DBF, deciduous broad‐leaved forest; Plant diversity, Simpson index of trees, shrubs and grass; Tem10, temperature of 10 cm depth; DSOC, dissolved soil organic carbon; TP, total phosphorus; TN, total nitrogen; Mo, moisture

The Mantel test indicated that soil temperature (Tem10) was significantly correlated (*p* < 0.01) with soil fungal diversity at both the taxonomic and functional levels (Table 2), followed by soil pH (*r* = 0.570, *p* < 0.001), Mo (*r* = 0.407, *p* < 0.001), and TN (*r* = 0.385, *p* < 0.001). Additionally, CCA resulted in models with a confidence level of *p* = 0.01 for both Illumina sequencing (Figure 5a) and GeoChip 4.0 data (Figure 5b). At the taxonomic level, soil Tem10 and soil Mo appeared to be important factors involved in shaping soil fungal diversity, and soil pH and TN were also important for the fungal community structure. At the fungal functional gene level, soil Tem10 had the longest projected length at the CCA axis 1, followed by soil Mo and TN. These results indicate that soil temperature and Mo may be the main factors involved in shaping soil fungal community structure, followed by soil pH and TN.

**Table 2:** Mantel test between ITS2 OTUs and functional genes of C and N cycling with environment factors

Environment factors	ITS2 OTUs		Functional genes	
	r	p	r	p
Plant diversity	0.168	0.076	0.314	0.018
Soil organic carbon	0.244	0.025	−0.001	0.405
Total nitrogen	0.385	0.001	0.048	0.322
Total phosphorus	0.149	0.108	0.061	0.302
pH	0.570	0.001	−0.047	0.597
Moisture	0.407	0.001	−0.008	0.459
Soil temperature of 10 cm depth	0.639	0.001	0.340	0.005

## 4 DISCUSSION

Soil fungal community structure and their dominant phyla have differed in previous studies (He et al., 2017; Shi et al., 2014; Zhao et al., 2016). In our study, soil fungal taxonomic community structure differed between EBF and DBF, and the phylum *Zygomycota* was the most abundant in both DBF and EBF. Chen et al. (2019) found that *Zygomycota* accounted for 45% of the phyla in primary stands of tropical rainforests. Some previous studies found that *Ascomycota* (Geml et al., 2014; He et al., 2017; Yang, Dou, Huang, & An, 2017) or *Basidiomycota* (Liu, Liu, Chen, Wang, & Zhang, 2018) were the dominant phyla in Andean Yungas forest, temperate deciduous forests and subtropical evergreen forests of eastern China, and Loess Plateau soil. *Zygomycota* are oligotrophic microbes (Zhao et al., 2016). EBF had high plant diversity and might have a higher ability to input soil nutrients than DBF due to the high abundance of *Zygomycota* (Zhao et al., 2016). *Mortierella* and *Umbelopsis* were the most abundant genera among the phylum *Zygomycota* in our study. *Umbelopsis* had the highest relative abundance in DBF and *Mortierella* had the highest relative abundance in EBF. However, these genera have the same function and are known to synthesize polyunsaturated fatty acids (Nyilasi et al., 2015). Shi et al. (2014) also found that *Mortierella* and *Umbelopsis* genera accounted for a large proportion of the soil fungal community. However, Zhao et al. (2016) reported that *Penicillium* and *Aspergillus* were the most prevalent soil fungal genera in middle subtropical forests.

Microbes play typical roles in regulating ecosystem C and N cycling, especially for soil fungi (Sun et al., 2015). It is a great challenge to establish relationships between soil microbial communities and the functional activity related to ecosystem function because of soil microbial diversity and the complexity of natural ecosystem (Zhang, Cong, et al., 2014). Many studies have described soil microbial community structure in various natural environments, but failed to identify key communities related to detailed ecosystem functional processes (Zhang, Cong, et al., 2014; López‐Lozano et al., 2013). GeoChip data are widely used to analyze environmental microbial functional diversity (Cong, Liu, et al., 2015; Zhang, Cong, et al., 2014) and estimate how soil C and N content changes with microbial functional gene diversity (Xue et al., 2016; Zhang et al., 2017). Although GeoChip is unable to directly reflect soil microbial functional activities, it can show the presence of genes that have functional capacity (Zhang, Cong, et al., 2014). In this study, many soil fungal functional gene relative intensities of both labile and recalcitrant C decomposition were significantly higher (*p* < 0.05) in EBF with gene relative intensities of C fixation being significantly lower (*p* = 0.027) in DBF. These results indicated soil fungal functional genes may play a large role in the turnover of soil C and N contents in EBF and DBF. Some previous studies also showed the similar results. For example, Xue et al. (2016) found that SOC content decreased as a result of increased bacterial C degradation genes, and soil N content was reduced with a higher abundance of *nifH*, *gdh*, *ureC,* and *nirK* genes. Zhang et al. (2017) suggested that SOC content decreased following an increase in the relative abundance of labile C decomposition genes after alpine meadow degeneration succession. Ding et al. (2015) also found that SOC content decreased following an increase in C decomposition genes of soil bacteria in DBF.

The fMENs visualized the soil fungal network structure in an effort to extract genes with high connectivity, simplifying the process of massive data analysis. In this study, no module hubs or connectors and none of the top five nodes were shared between the two C cycling gene networks. DBF had higher modularity and more positive links than EBF, which may reflect higher resistance ability (Scheffer et al., 2012) and mutualism (Wu et al., 2016; Zhang, Zhao, Dai, Jiao, & Herndl, 2014). However, EBF appeared more complicated according to the higher average degree and clustering coefficient (Deng et al., 2012). Therefore, soil fungal diversity had the same trend with modularity distribution, but network complexity may have been induced by niche differentiation (Wu et al., 2016).

Temperature and moisture are the primary drivers in ecological processes (Brockett, Prescott, & Grayston, 2012; Cong, Yang, et al., 2015) and have significantly influenced species diversity of plants, animals, and microbes (Bell et al., 2009). In this study, the relative abundance of C cycling genes was significantly correlated with both soil temperature and moisture, consistent with the results reported by Zumsteg et al. (2013). Previous studies have suggested that warmer conditions significantly decrease the abundance of soil fungal biomarkers (Frey, Drijber, Smith, & Melillo, 2008), and that C utilization ability is higher in colder conditions (Bell et al., 2009). In theory, more soil substrates are used with increased soil temperature, resulting in decreased availability of soil substrates (Dijkstra et al., 2011; Kirschbaum, 2004). However, adverse responses have been reported in some previous studies (Newsham et al., 2015; Zhou et al., 2016). For example, Carey et al. (2016) suggested that increasing temperature had no significant correlation with microbial respiration. This may be because of substrate complexity, the inherent decomposability of microorganisms (Frey et al., 2013) or highly variable environments.

## 5 CONCLUSIONS

In summary, the soil fungal diversity in broad‐leaved forests differed significantly between EBF and DBF at both the taxonomic and functional levels. The relative abundance of many genes related to labile C degradation was significantly higher (*p* < 0.05) in DBF than EBF, and the relative gene abundance involved in C cycling was significantly negatively correlated with soil labile organic C. Molecular ecological network analysis revealed that the interaction and complexity among functional genes differed between EBF and DBF. Therefore, the soil fungal community structure and potential metabolic activity showed marked divergence between different broad‐leaved forest types, and the higher relative abundance of genes involved in C and N cycling in DBF would most likely cause soil C and N release or loss.

## CONFLICT OF INTERESTS

The authors declare that they have no competing interests.

## AUTHOR CONTRIBUTIONS

Y.Z. developed and framed research questions. Y.S., J.C., H. L., D. L., L.Y, and Q. L. finished the plant survey and collected data used in this analysis. Y. S. analyzed the data and wrote the first draft of the manuscript, and all authors contributed substantially to revisions.

## ETHICS STATEMENT

None required.

## Data Availability

The sequencing datasets analyzed during the current study are available in the GenBank database with accession number of SRP115169.

## APPENDIX 1

**Table A1:** Proportion of genera which more than 1% at least in one site

Phyla	Genera name	EBF	DBF	*p* value
*Ascomycota*	*Cortinarius*	0.05 ± 0.11	3.19 ± 5.01	0.120
	*Davidiella*	1.07 ± 1.39	0.07 ± 0.04	0.082
	*Phoma*	7.37 ± 7.45	0.63 ± 0.51	0.038
	*Pseudogymnoascus*	3.28 ± 4.49	9.90 ± 15.85	0.275
	*Trichoderma*	4.08 ± 6.86	0.73 ± 0.90	0.211
*Basidiomycota*	*Cryptococcus*	0.38 ± 0.45	1.16 ± 1.13	0.101
	*Laccaria*	0.00	1.05 ± 1.27	0.052
	*Russula*	0.58 ± 1.04	4.51 ± 5.61	0.090
*Zygomycota*	*Mortierella*	44.07 ± 29.79	8.30 ± 5.73	0.011
	*Umbelopsis*	2.25 ± 2.95	49.20 ± 26.46	0.001
*Unclassified*	*Unclassified*	11.98 ± 7.09	6.51 ± 8.63	0.188

Note

Data presented above were percentage of each genus which had an account more than 1% at least one site, unclassified genera in each phylum were not showed, *P* values were measured through unpaired *t* test.

**Table A2:** Dissimilarity tests of soil fungal community structure detected by Illumina sequencing

Distance type	MRPP		ANOSIM		Adonis	
	Δ	*p*	*R*	*p*	*R* ^2^	*p*
Bray–curtis	0.685	0.001	0.784	0.001	0.330	0.001
Euclidean	3,265.073	0.001	0.454	0.001	0.281	0.001

Note

Three different permutation tests were conducted, including MRPP (Multiple Response Permutation Procedure), ANOSIM (Analysis of similarities), and Adonis (Permutational Multivariate Analysis of Variance).

**Table A3:** Pearson correlation matrix between soil nitrogen fractions with fungal nitrogen cycling genes

	*ureC*	*gdh*	*nitrate reductase*	*glnA*	*nirK*	*p450nor*	Total nitrogen	Available nitrogen
*ureC*	1.00	−0.61*	0.61*	−0.72**	−0.64**	0.53*	0.23	0.16
*gdh*		1.00	−0.13	0.61*	0.52*	−0.27	−0.55*	−0.52*
*nitrate reductase*			1.00	−0.45	−0.20	0.19	−0.08	−0.04
*glnA*				1.00	0.30	−0.50	−0.46	−0.41
*nirK*					1.00	−0.54*	−0.22	−0.22
*p450nor*						1.00	0.38	0.34
Total nitrogen							1.00	0.97**
Available nitrogen								1.00

Note

Significant difference was labeled with ^*^(*p* < 0.05) or ^**^(*p* < 0.01) above corresponding numbers.

**Table A4:** Topological properties of the empirical functional molecular ecological networks of fungal communities and their associated random

Community	Empirical networks								Random networks		
	Threshold	Total nodes	Positive interactions	*R* ^2^ of power‐law	Average degree (avgK)	Average clustering coefficient (avgCC)	Average path distance (GD)	Modularity	Average clustering coefficient (avgCC)	Average path distance (GD)	Modularity
EBF	0.980	1,428	73.93%	0.822	19.987	0.423	4.035	0.620	0.061 ± 0.002	2.888 ± 0.009	0.166 ± 0.002
DBF	0.970	1,317	93.37%	0.779	5.585	0.323	5.037	0.794	0.102 ± 0.004	3.354 ± 0.019	0.386 ± 0.003

**Table A5:** The top five nodes with high connectivity of C degradation cycling gene networks

ID	Node degree	Gene name	Subcategory2
In EBF			
238592310	135	*exoglucanase*	Cellulose
261204715	123	*glx*	Lignin
39968417	121	*cutinase*	cutin
242217049	114	*ara*	Hemicellulose
145238722	114	*exoglucanase*	Cellulose
In DBF			
50952845	272	*mnp*	Lignin
147225254	175	*protease erine*	protein
310799874	170	*exopolygalacturonase*	Pectin
302418354	169	*hmgC*	Others
67901318	160	*pec CDeg*	Pectin

**Table A6:** Module hubs of C degradation cycling gene networks

ID	Gene name	Subcategory2
In EBF (23)		
58270684	*AceA*	Others
261197303	*AceA*	Others
119483664	*alpha galactosidase*	Others
284181765	*cellobiase*	Cellulose
295673792	*cellobiase*	Cellulose
238843148	*cutinase*	cutin
302882620	*cutinase*	cutin
39968417	*cutinase*	cutin
88184083	*endochitinase*	Chitin
225677854	*endochitinase*	Chitin
151336975	*endochitinase*	Chitin
115390094	*endochitinase*	Chitin
201066455	*endoglucanase*	Cellulose
310790415	*endopolygalacturonase*	Pectin
238592310	*exoglucanase*	Cellulose
146424871	*exoglucanase*	Cellulose
115397527	*glucoamylase*	Starch
261204715	glx	Lignin
46124631	*phospholipase C*	lipids
145573242	*protease serine*	protein
145583579	*protease serine*	protein
295673568	*Sulfhydryl oxidase*	Others
302675262	*xylanase*	Hemicellulose
In DBF (31)		
67904096	*alpha galactosidase*	Others
145228347	*ara*	Hemicellulose
565664	*cellobiase*	Cellulose
310791430	*cutinase*	cutin
302414708	*dextranase*	Others
117582119	*endochitinase*	Chitin
1223924	*endochitinase*	Chitin
2967835	*endopolygalacturonase*	Pectin
46241266	*exoglucanase*	Cellulose
294196	*exoglucanase*	Cellulose
310799874	*exopolygalacturonase*	Pectin
302682011	*exopolygalacturonase*	Pectin
34392447	*glucoamylase*	Starch
169851372	*glx*	Lignin
302418354	*hmgC*	Others
267850581	*lip*	Lignin
66130215	*lip*	Lignin
197260976	*mannanase*	Hemicellulose
50952845	*mnp*	Lignin
67901318	*pec CDeg*	Pectin
302673816	*pec CDeg*	Pectin
189189022	*pectinase*	Pectin
121713452	*pel CDeg*	Pectin
58176536	*phenol oxidase*	Lignin
32399641	*phenol oxidase*	Lignin
126105515	*phenol oxidase*	Lignin
209978146	*phospholipase A2*	lipids
71001696	*phospholipase A2*	lipids
116196714	*phospholipase D*	lipids
147225254	*protease serine*	protein
145253010	*vdh*	Others
